# Supplementary material for: The elongation of Mest transcript into MestXL sustains, but does not initiate, the maternal allele bias of its convergent gene Copg2 during neurogenesis
Source: Nat Commun. 2026 Sep 23;17:11045. doi: 10.1038/s41467-026-77877-3 (PMC13691114; doi:10.1038/s41467-026-77877-3)
Supplement: Supplementary file 1 [file 41467_2026_77877_MOESM1_ESM.pdf]

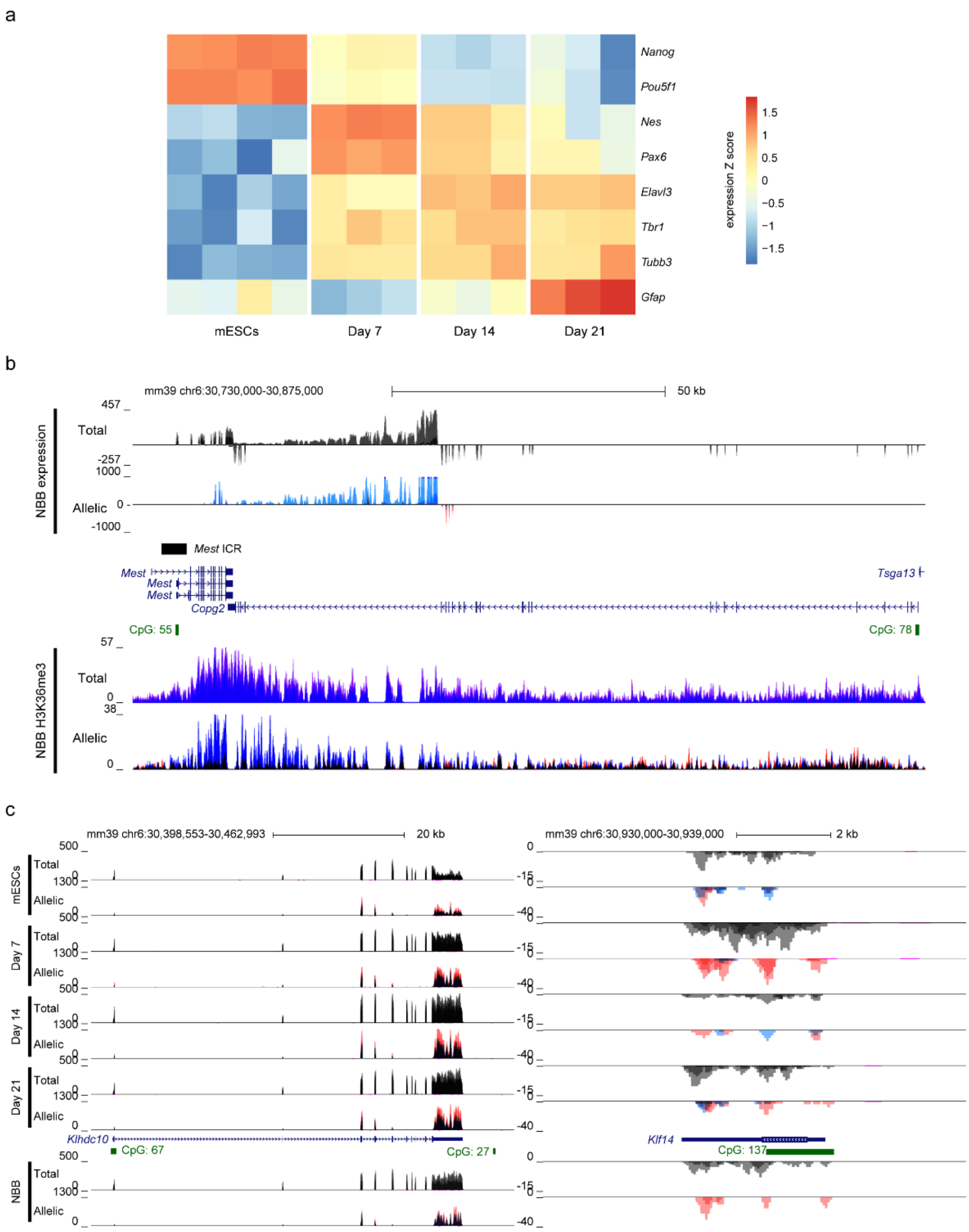

Supplementary Figure 1 Differentiation and *Mest* imprinted domain dynamics during mouse brain organoid development.

a Heatmap illustrating the expression levels of specific markers, as measured by RNA-seq, during the differentiation of brain organoids. Pluripotency markers (*Nanog*, *Pou5f1*), neural progenitor markers (*Nestin*, *Pax6*), neuronal markers (*Elavl3*, *Tbr1*, *Tubb3*), and the astrocyte marker *Gfap* were analyzed across B/J embryonic stem cells (ESCs) and at days 7, 14, and 21 of brain organoid differentiation. Data are presented for each independent experiment, with sample sizes (biological replicates) of n=4 for ESCs and n=3 for days 7, 14, and 21. The color scale represents Z-score expression levels. **b** Genome browser view of the *Mest* and *Copg2* loci, showing allelic RNA-seq signals (upper panel) and H3K36me3 enrichment obtained by Cut&Run (lower panel) in merged B/J and J/B newborn brain tissue (NBB) (n = 2 biological replicates for RNA-seq; n = 4 biological replicates for C&R). For each condition, the quantitative and merged parental allelic signals are at the top and bottom, respectively. Maternal and paternal expression levels are shown in red and blue, respectively. **c** Genome browser view at the *Klhdc10* and *Kfl14* genes, respectively, to show the allelic-oriented RNA-seq signals in B/J ESCs (n=4) and at day 7 (n = 3), day 14 (n = 3), and day 21 (n = 3) of independent in vitro brain organoid differentiation, as well as in merged B/J and J/B newborn brain tissue (NBB) (n = 2). For each condition, the quantitative and merged parental allelic RNA-seq signals are at the top and bottom, respectively. Maternal and paternal expression levels are shown in red and blue, respectively.

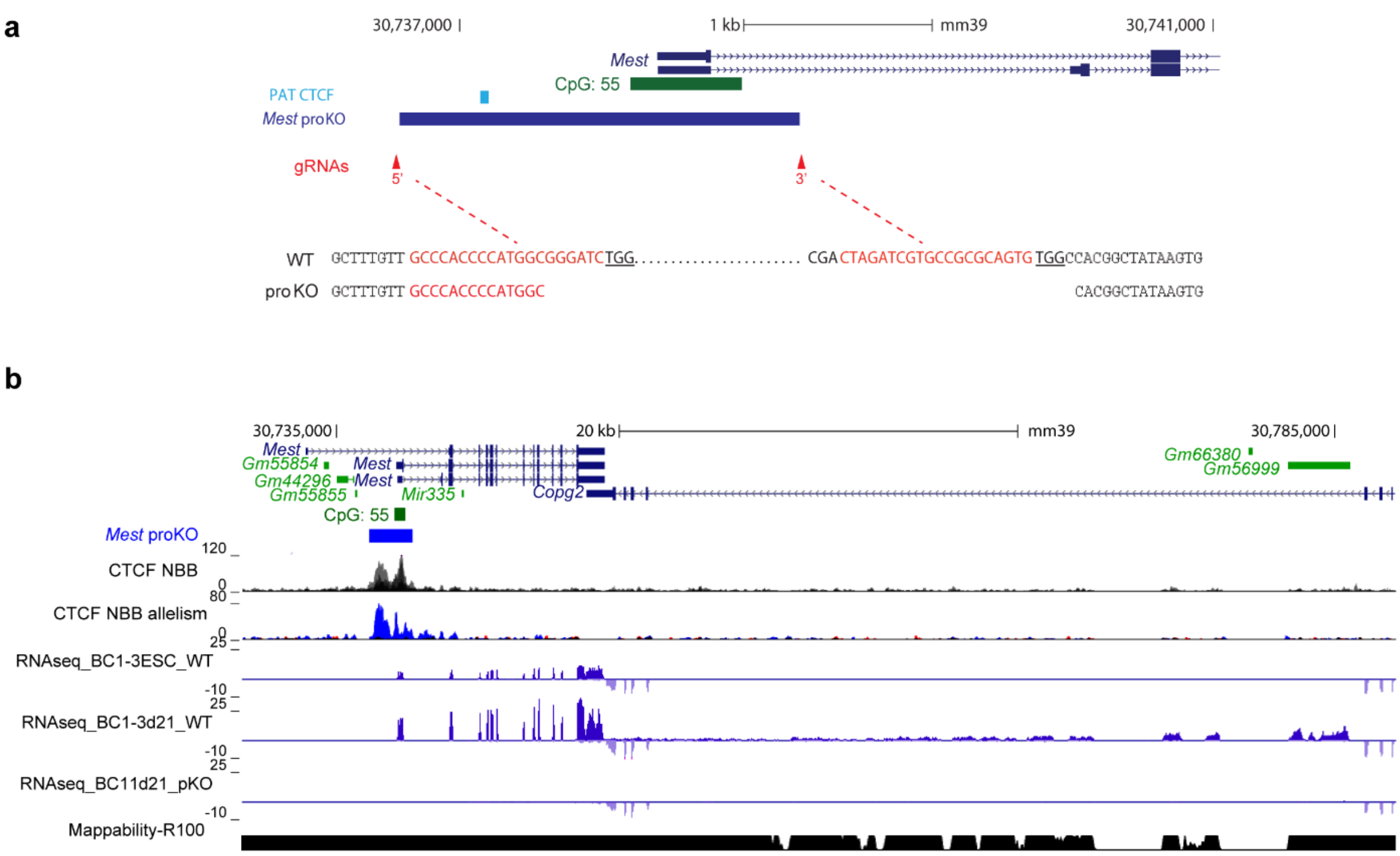

Supplementary Figure 2: Deletion of the *Mest* canonical promoter eliminates *MestXL* expression in brain organoids

a Top- Screenshot of a UCSC Genome Browser window from mouse genome build mm39 showing the 5’ end of the *Mest* gene with its exon 1-associated CpG island, the position of the upstream CTCF binding site on the paternal allele and the extent of the 2131-bp *Mest* promoter deletion (*Mest* ProKo), defined by unique 5’ and 3’ gRNA for CRISPR-Cas9 deletion. Bottom-Alignment of the DNA sequences of the WT and *Mest* proKO line alleles, with the position of CRISPR gRNA sequences shown in red and PAMs in bold/underlined characters.

b UCSC Genome Browser session centered on the *Mest*-*Copg2* region, annotated with BED tracks as in A, and showing: i) CTCF C&R tracks from newborn brains (NBB) with quantitative and merged parental allelic signals displayed in the upper and lower panels, respectively. Maternal and paternal enrichments are shown in red and blue, respectively.; ii) bulk RNA-seq tracks for ESCs and d21 organoids from the WT parental line BC1.3, and d21 organoids from the Mest+/proKO ESC BC11 (pKO).

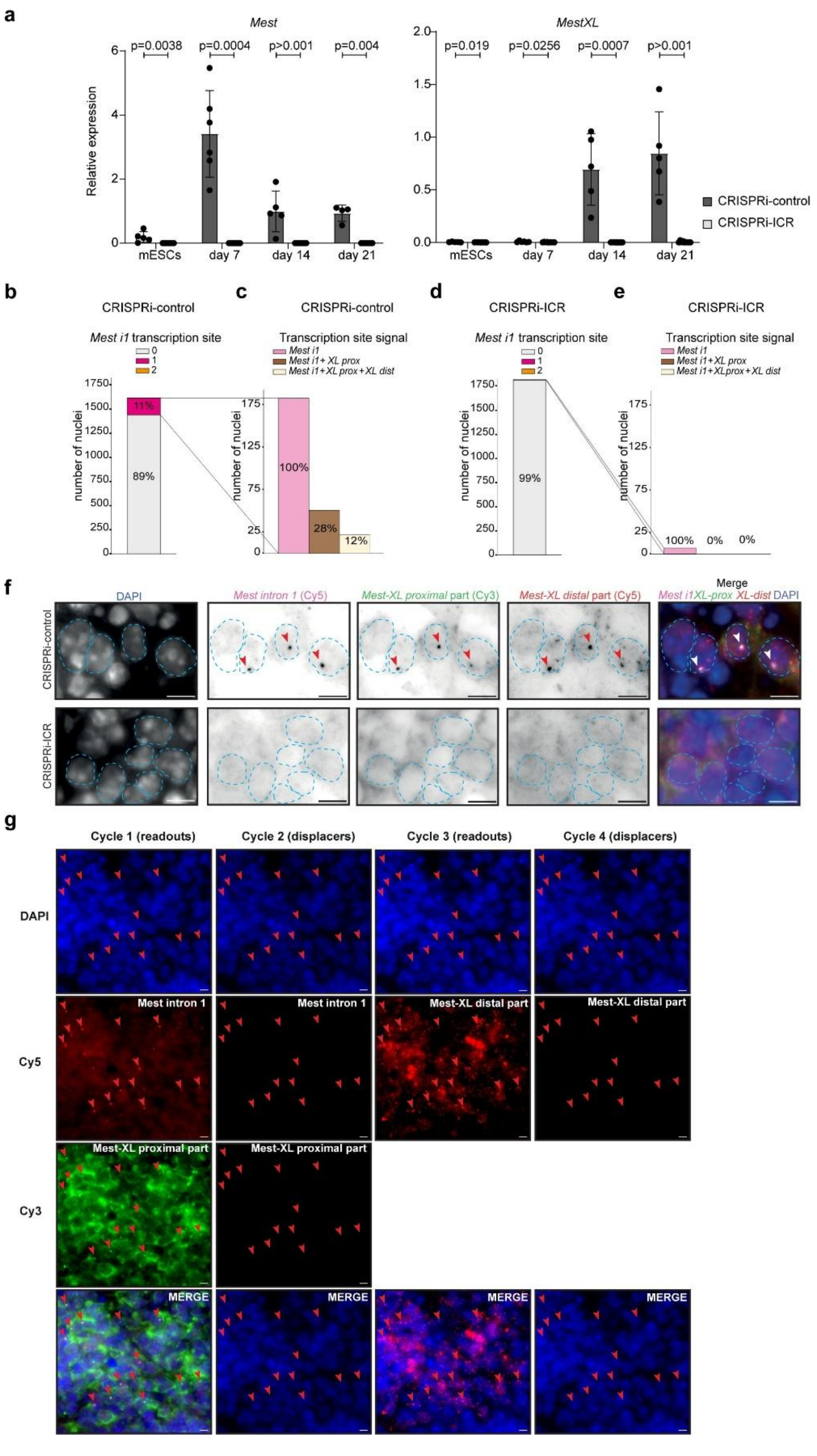

Supplementary Figure 3: Alteration of the *Mest* canonical promoter eliminates *MestXL* expression in brain organoids

a RT-qPCR analysis of *Mest* and *MestXL* expression in CRISPRi-ICR and CRISPRi-control B/J ESCs (n=5 biological replicates), and at Days 7, 14, and 21 (n=5 each) of organoid differentiation. Expression is shown as a percentage relative to the geometric mean of three housekeeping genes (*Gapdh*, *Gus*, *Tbp*), mean ± SD. Significance was assessed by two-sided Mann-Whitney U test, p value is shown in the figure. **b** Quantification of the number of nuclei showing no, one, or two transcription sites using the *Mest* intronic probe (*Mest i1*) in CRISPRi-control day 14 brain organoids (N=1620 nuclei). **c** Quantification of positive nuclei for *Mest i1* displaying also spots co-localised for *MestXL proximal* part and *proximal* + *distal* part (*XL prox* and *XL dist*) in CRISPRi-control day 14 brain organoids. **d**, **e** Same as b and c but on CRISPRi-ICR day 14 brain organoids (N=1824 nuclei). **d**, **e** Same than b and c but on CRISPRi-ICR day 14 brain organoids (N=1824 nuclei). f Sequential smiFISH experiments in CRISPRi-control (top) and CRISPRi-ICR (bottom) day 14 brain organoid cryosection

with probes targeting the intron 1 of the *Mest* transcript (magenta) and *proximal* (green) and *distal* (red) regions of the *MestXL* transcript. Red arrowheads report the position of the *Mest* nascent transcript (intronic probe) in the different rounds of imaging. Images are maximum-intensity projections of 5 planes. Merged images are shown on the right. Nuclei are indicated by blue dashed lines from DAPI images. Scale bars: 5 µm. **g** Washout cycles of sequential smiFISH on a CRISPRi-control Day 14 organoid cryosection. Cycle 1: Mest intron1 (red, Cy5) and MestXL proximal (green, Cy3); both displaced/bleached by Cycle 2 (note absence of signal). Cycle 3: MestXL distal (red, Cy5), displaced/bleached by Cycle 4. Red arrowheads mark the Mest nascent transcript (intronic probe) across rounds. Images (max-intensity projections, 5 planes) shown at the same intensity scale. Scale bars: 5 µm. Source data for panel 3a are in the Source Data file.

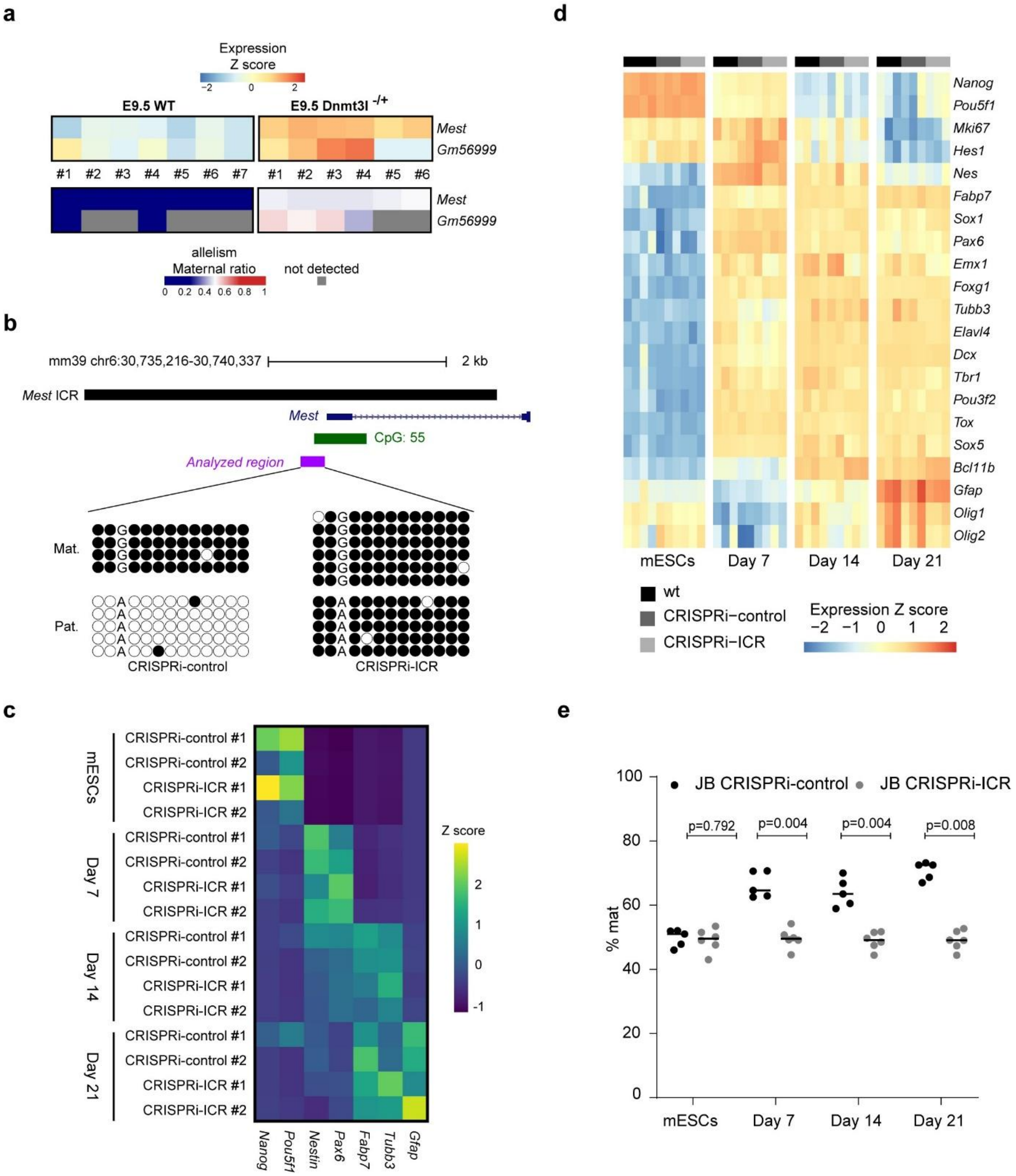

Supplementary Figure 4: Increased *Copg2* expression in brain organoids derived from CRISPRi-ICR embryonic stem cells

a Heatmap showing the expression (upper panel) and allelic (lower panel) levels of *Mest* and *MestXL* (assessed through the transcript annotation Gm56999) in E9.5 WT (left panel) and Dnmt3l$^{-/+}$ embryos; Data are presented for each independent embryo (WT: n=7; Dnmt3l$^{-/+}$: n=6). Insufficient signal at informative allelic sites was detected in some *Dnmt3L*$^{-/+}$ embryos to establish an allelic ratio of Gm56999. **b** Schematic of the mouse *Mest* promoter region, illustrating CpG islands (CGI) as annotated in the UCSC Genome Browser. The region analyzed by bisulfite sequencing is marked in purple. Bisulfite sequencing data from CRISPRi-ICR and CRISPRi-control J/B ES cells are shown below, with each row representing CpG dinucleotides on a single chromosome. Filled and open circles indicate methylated and unmethylated CpGs, respectively. Parental origin (Mat., maternal; Pat., paternal) was determined using a strain-specific SNP. **c** Expression levels of pluripotency and neural lineage markers, assessed by RT-qPCR, in two independent clones of CRISPRi-ICR and CRISPRi-control ESCs and at days 7, 14, and 21 of *in vitro* brain organoid differentiation. **d** Expression levels of pluripotency and neural markers in WT and CRISPRi-ICR ESCs (n = 4 biological replicates) and at days 7 (n = 3

biological replicates), 14 (n = 3 biological replicates), and 21 (n = 3 biological replicates) of *in vitro* brain organoid differentiation. **e** Percentage of *Copg2* maternal expression measured in CRISPRi-ICR (n=6 biological replicates) and CRISPRi-control (n=5 biological replicates J/B embryonic stem cells (ESCs), as well as at days 7, 14, and 21 of *in vitro* brain organoid differentiation. Values were derived from sequencing of RT-PCR products, followed by quantification of ABI-format sequence files. Statistical significance was determined with a two-sided Mann-Whitney U test, p value is shown in the figure. Source data for panels 4c and 4e are provided as a Source Data file.

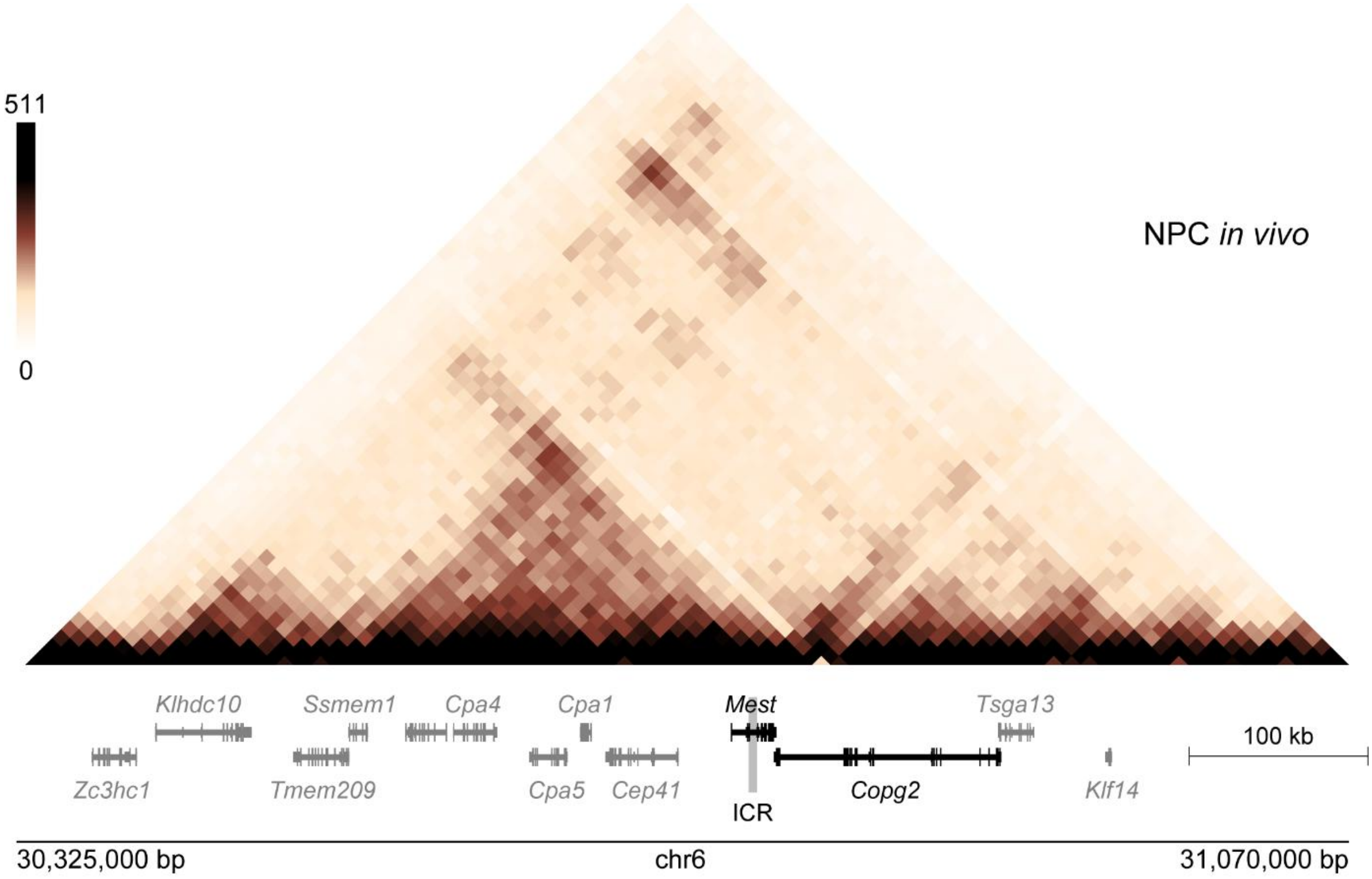

Supplementary Figure 5: Two chromatin subdomains anchored at the *Mest* ICR

A) Genome Browser view of the *Mest/Copg2* domain showing reanalyzed Hi-C data in in vivo NPCs.

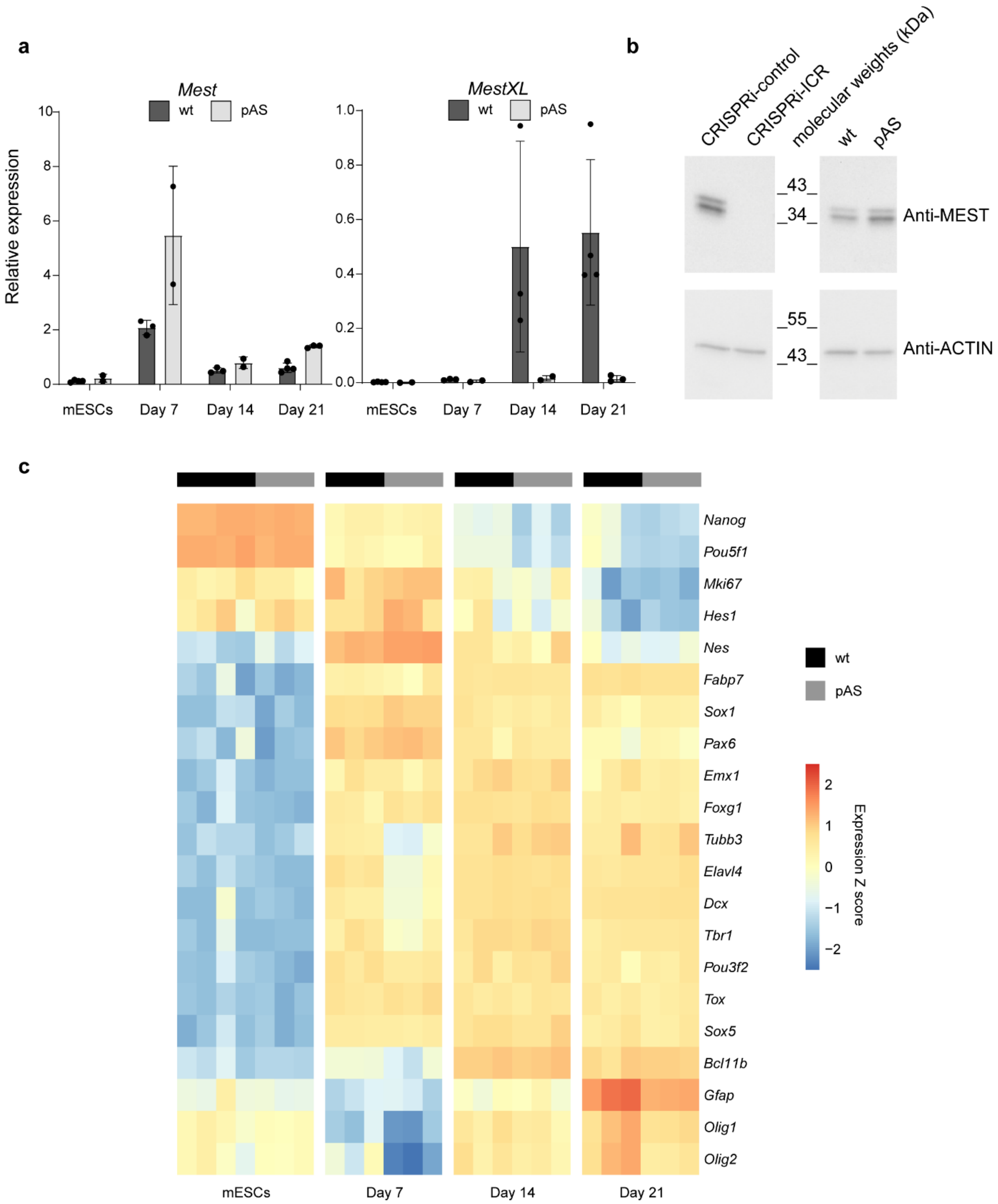

Supplementary Figure 6: *Mestxl*, but not *Mest,* expression is abolished in pAS cells

a RT-qPCR analysis of *Mest* and *MestXL* expression levels in ES-pAS cells and at Day 7, Day 14, and Day 21 of in vitro brain organoid differentiation. Values represent the mean of three independent experiments, each analyzed in duplicate. Data are presented as a percentage of expression relative to the geometric mean of three housekeeping genes (*Gapdh*, *Gus*, and *Tbp*), and are shown as mean ± SD. **b** Western blot analysis of *Mest* (upper panel) and β-actin (lower panel) in WT and pAS cells at Day 7. Similar analyses performed in CRISPRi-control and CRISPRi-ICR cells, where *Mest*/MEST expression is abolished, confirm the specificity of the antibody. **c** Expression levels of pluripotency and neural markers in WT and pAS ESCs (n = 4) and at days 7 (n = 3), 14 (n = 3), and 21 (n = 3) of independent *in vitro* brain organoid differentiation. Source data for panels 6a and 6b are provided as a Source Data file.
