# Supplementary material for: The elongation of Mest transcript into MestXL sustains, but does not initiate, the maternal allele bias of its convergent gene Copg2 during neurogenesis
Source: Nat Commun. 2026 Sep 23;17:11045. doi: 10.1038/s41467-026-77877-3 (PMC13691114; doi:10.1038/s41467-026-77877-3)
Supplement: Supplementary file 2 [file 41467_2026_77877_MOESM2_ESM.pdf]

Description of Additional Supplementary Files

Supplementary Data 1: This file contains information on primers.

Supplementary Data 2: This file contains information on antibodies.

Supplementary Data 3: This file contains information on smiFISH probes.
