# Supplementary material for: The elongation of Mest transcript into MestXL sustains, but does not initiate, the maternal allele bias of its convergent gene Copg2 during neurogenesis
Source: Nat Commun. 2026 Sep 23;17:11045. doi: 10.1038/s41467-026-77877-3 (PMC13691114; doi:10.1038/s41467-026-77877-3)
Supplement: Supplementary file 3 [file 41467_2026_77877_MOESM6_ESM.pdf]

nature portfolio

Corresponding author(s): T. Bouschet, F. Court, P.Arnaud

Last updated by author(s): August 27, 2026

Reporting Summary

Nature Portfolio wishes to improve the reproducibility of the work that we publish. This form provides structure for consistency and transparency in reporting. For further information on Nature Portfolio policies, see our Editorial Policies and the Editorial Policy Checklist.

Statistics

For all statistical analyses, confirm that the following items are present in the figure legend, table legend, main text, or Methods section.

n/a	Confirmed	
☐	☒	The exact sample size (*n*) for each experimental group/condition, given as a discrete number and unit of measurement
☐	☒	A statement on whether measurements were taken from distinct samples or whether the same sample was measured repeatedly
☐	☒	The statistical test(s) used AND whether they are one- or two-sided<br>*Only common tests should be described solely by name; describe more complex techniques in the Methods section.*
☒	☐	A description of all covariates tested
☒	☐	A description of any assumptions or corrections, such as tests of normality and adjustment for multiple comparisons
☐	☒	A full description of the statistical parameters including central tendency (e.g. means) or other basic estimates (e.g. regression coefficient) AND variation (e.g. standard deviation) or associated estimates of uncertainty (e.g. confidence intervals)
☐	☒	For null hypothesis testing, the test statistic (e.g. *F*, *t*, *r*) with confidence intervals, effect sizes, degrees of freedom and *P* value noted<br>*Give P values as exact values whenever suitable.*
☒	☐	For Bayesian analysis, information on the choice of priors and Markov chain Monte Carlo settings
☒	☐	For hierarchical and complex designs, identification of the appropriate level for tests and full reporting of outcomes
☒	☐	Estimates of effect sizes (e.g. Cohen's *d*, Pearson's *r*), indicating how they were calculated

Our web collection on statistics for biologists contains articles on many of the points above.

Software and code

Policy information about availability of computer code

Data collection	Sanger sequencing data were analyzed using Finch TV.<br>qPCR data were processed with LightCycler 480 Software (Release 1.5.1.62).<br>Allelic ratios were determined using Mutation Surveyor DNA variant analysis software.<br>Sequential imaging was performed using SRX 7.6.27 software available with the Vutara VXL imaging station (Bruker).<br>Fluorescent imaging was conducted on an AxioImager Z1 (Zeiss) with ZEN 3.8 Blue Edition software.<br>Confocal imaging was performed on an LSM980 AIRYSCAN using ZEN 3.4 (Blue Edition) software (Zeiss).<br>Western blots were imaged using Bio-Rad Image Lab Touch software (v2.2.0.08) on a ChemiDoc apparatus (Bio-Rad).
Data analysis	The Methods section details all the open-source software tools that were used to process the next-generation sequencing data analyzed in the study (WGBS,RNA seq, C&R; HicCapture, Atac seq, long read). They are as follows:<br>Bismark<br>TrimGalore (v 0.6.10)<br>Hisat2 (version 2.2.1)<br>samtools (version 1.21)<br>SNPsplit (version 0.4.0)<br>bamCoverage (version 3.5.6)<br>htseq-count version 2.0.9)<br>bedtools maskfasta tool (version 2.31.1)

IsoQuant (version 3.4.1)
HICUP (version 0.9.2)
R (version 4.5.2)
HiC-Pro (version 3.1.0).
Bowtie2 (version 2.5.4)
macs2 (version 2.1.4)

For sequential smi-FISH experiments, the following sofwares were used:
Cellpose(version 4.0.7)
Big-fish (version 0.6.2)
Small Fish (version 1.0) 10.5281/zenodo.15683712
The pipeline is available here:
https://doi.org/10.5281/zenodo.15683712

For manuscripts utilizing custom algorithms or software that are central to the research but not yet described in published literature, software must be made available to editors and reviewers. We strongly encourage code deposition in a community repository (e.g. GitHub). See the Nature Portfolio guidelines for submitting code & software for further information.

Data

Policy information about availability of data

All manuscripts must include a data availability statement. This statement should provide the following information, where applicable:

- Accession codes, unique identifiers, or web links for publicly available datasets
- A description of any restrictions on data availability
- For clinical datasets or third party data, please ensure that the statement adheres to our policy

The RNA-seq data generated in this study have been deposited in the GEO data repository database under accession codes GSE324877 [https://www.ncbi.nlm.nih.gov/geo/query/acc.cgi?acc=GSE324877], GSE325581 [https://www.ncbi.nlm.nih.gov/geo/query/acc.cgi?acc=GSE325581] and GSE325582 [https://www.ncbi.nlm.nih.gov/geo/query/acc.cgi?acc=GSE325582].
The Hi-C capture data generated in this study have been deposited in the GEO data repository database under accession code GSE325578 [https://www.ncbi.nlm.nih.gov/geo/query/acc.cgi?acc=GSE325578].
The Cut&Run data generated in this study have been deposited in the GEO data repository database under accession code GSE325583 [https://www.ncbi.nlm.nih.gov/geo/query/acc.cgi?acc=GSE325583].
ESC and NPC whole-genome bisulfite sequencing (WGBS) data were obtained from GEO DataSets under the accession numbers GEO: GSM748786 [https://www.ncbi.nlm.nih.gov/geo/query/acc.cgi?acc=GSM748786] and GEO: GSM748788, [https://www.ncbi.nlm.nih.gov/geo/query/acc.cgi?acc=GSM748788] respectively.
Long-read sequencing from mouse cortex were obtained from SRA repository under the project number: PRJNA663877 (8 months cortices : SRR12653624 [https://www.ncbi.nlm.nih.gov/sra/?term=SRR12653624], SRR12653626 [https://www.ncbi.nlm.nih.gov/sra/?term=SRR12653626], SRR12653628 [https://www.ncbi.nlm.nih.gov/sra/?term=SRR12653628]).
Hi-C data for NPC purified in-vivo from E14.5 neocortex were obtained from the GEO: GSE96107 DataSet [https://www.ncbi.nlm.nih.gov/geo/query/acc.cgi?acc=GSE96107].

Mouse reference genome mm39 with GENCODE vM37 basic annotation were used for data analysis in this study.

Research involving human participants, their data, or biological material

Policy information about studies with human participants or human data. See also policy information about sex, gender (identity/presentation), and sexual orientation and race, ethnicity and racism.

Reporting on sex and gender	N/A
Reporting on race, ethnicity, or other socially relevant groupings	N/A
Population characteristics	N/A
Recruitment	N/A
Ethics oversight	N/A

Note that full information on the approval of the study protocol must also be provided in the manuscript.

Field-specific reporting

Please select the one below that is the best fit for your research. If you are not sure, read the appropriate sections before making your selection.

[x] Life sciences [ ] Behavioural & social sciences [ ] Ecological, evolutionary & environmental sciences

Life sciences study design

All studies must disclose on these points even when the disclosure is negative.

Sample size

No statistical method was used to predetermine sample size. Our experimental design followed community standards, with a minimum of three independent biological replicates, each including at least two technical replicates, per experiment to enable statistical analysis. Omics approaches were replicated to ensure adequate genome coverage.

Data exclusions

In Seq-smiFISH analyses, the following filtering criteria were applied prior to quantification
Spots detected on first 5 slices were discarded due to noisy signal.
Nuclei with volume inferior to 10 000 px were discarded
Any cell whose mask lies on the border of the field of view was considered incomplete and thus was discarded.
Any spot detected outside of cell segmentations was discarded.

Replication

Cut&Run and Hi-C Capture experiments were performed in two biological replicates.
RNA-seq was performed in 3 to 4 biological replicates in B6×JF1 WT, pAS, and CRISPi-ICR ES cell lines (see figure legends for details), and in 3 biological replicates at days 7, 14, and 21 of brain organoid differentiation.
RNA-seq was also performed in 7 and 6 biological replicates in WT and Dnmt3L-/+ E9.5 embryos respectively, in 2 biological replicates in newborn brain, and in one replicate in the pKO cell line.
RT-qPCR and ChIP-qPCR were performed in 3 to 6 and 3 biological replicates respectively (see figure legends for details)

Randomization

Randomization was not applicable, as experimental groups were defined by fixed genetic or genotypic parameters intrinsic to the biological material (Dnmt3L genotype, CRISPRi-control versus CRISPRi-ICR cell line, pAS line or differentiation stage). These categories are non-interchangeable and therefore cannot be randomly assigned.

Blinding

Blinding was not performed because the study relies exclusively on objective, quantitative molecular readouts (allele-specific RNA-seq, RT-qPCR, ChIP-qPCR, CUT&RUN, Hi-C capture, and smiFISH quantification), generated and analyzed through automated instrumentation and computational pipelines based on strain-specific SNPs. Sample identity does not influence signal acquisition or the algorithms used for quantification, precluding observer bias.

Reporting for specific materials, systems and methods

We require information from authors about some types of materials, experimental systems and methods used in many studies. Here, indicate whether each material, system or method listed is relevant to your study. If you are not sure if a list item applies to your research, read the appropriate section before selecting a response.

Materials & experimental systems

n/a	Involved in the study	
☐	☒	Antibodies
☐	☒	Eukaryotic cell lines
☒	☐	Palaeontology and archaeology
☐	☒	Animals and other organisms
☒	☐	Clinical data
☒	☐	Dual use research of concern
☒	☐	Plants

Methods

n/a	Involved in the study	
☐	☒	ChIP-seq
☒	☐	Flow cytometry
☒	☐	MRI-based neuroimaging

Antibodies

Antibodies used

Anti-Chicken IgY (IgG) Sigma-Aldrich Polyclonal ref: C2288 lot: 23M4839 1 µg/reaction
anti-H3K9ac Merck Millipore Polyclonal ref: 06 -942 lot: #2511087; #31636 1 µg/reaction
anti-H3K4me2 Abcam Monoclonal ref; Ab32356 lot: GR 253788-9; YI06150152 1 µg/reaction
anti-H3K4me3 Diagenode Polyclonal ref: C15410003 lot: A8034D 1 µg/reaction
anti-H3K27ac Abcam Polyclonal ref: Ab4729 lot:1101610-4 1 µg/reaction
anti-H3K27me3 Merck Millipore Polyclonal ref: 07-449 D lot: AM1588246 1 µg/reaction

anti-H3K4me3 Epicypher Mixed monoclonal ref: 13-0041 lot:24213007-97 0.5 µg/reaction
anti-H3K27ac Abcam Polyclonal ref: Ab4729 lot:1101610-4 0.5 µg/reaction
anti-CTCF Diagenode Monoclonal ref: C15410210 lot: A2354-00234P 0.5 µg/reaction
anti-H3K27me3 Diagenode Monoclonal ref: C15410195 lot:A824D 0.5 µg/reaction
Anti-Rabbit IgY (IgG) Epicypher Polyclonal ref: 13-0042 lot:24213007-87 0.5 µg/reaction

Anti-NESTIN Santa Cruz Monoclonal Mouse ref: sc-33677 lot:B2515 1/1000
Anti-PAX6 Covance Polyclonal Rabbit ref:PRB-278P lot:B201255 1/2000
Anti-TUBB3 Covance Monoclonal Mouse ref:MMS-435P lot: B235555 1/1000

Anti-FABP7 Cell Signalling Monoclonal Rabbit	ref:13347	lot: 1	1/1000
Anti-ELAVL3 Santa Cruz Monoclonal Mouse	ref:sc-515624	lot:G2519	1/1000
Anti-TBR1 Cell Signalling Monoclonal Rabbit	ref: 49661	lot:1	1/2000
Anti-MEST Abcam Monoclonal Rabbit	ref: ab151564	lot:1076724-8	1/1000
Anti-ACTIN Chemicon Monoclonal Mouse	ref: MAB1501	lot: 2653087	1/5000

Validation

All antibodies used in this study are commercially available and have been validated by their respective providers for the intended applications (see provider websites for details)

Eukaryotic cell lines

Policy information about cell lines and Sex and Gender in Research

Cell line source(s)

The hybrid male embryonic stem cell (ESC) lines were previously derived from blastocysts obtained from reciprocal crosses between C57BL/J (B) and JF1 (J) mice (Montibus et al.,2021). Among these cell lines, B/J E2 was used by the authors to derived pAS, CRISPi-ICR and pKo cell lines in this current study

Authentication

Cell lines were not authenticated by whole genome sequencing. However, their allelic transcriptome and capacity for proliferation and differentiation into brain organoids confirm their integrity.

Mycoplasma contamination

Cell lines are regularly tested for mycoplasma contamination by an external provider (Eurofins). All cell lines used in this study tested negative for mycoplasma.

Commonly misidentified lines
(See ICLAC register)

No commonly misidentified cell lines were used in this study.

Animals and other research organisms

Policy information about studies involving animals; ARRIVE guidelines recommended for reporting animal research, and Sex and Gender in Research

Laboratory animals

Newborn brains were collected from 1- to 3-day-old mice obtained from reciprocal crosses between Mus musculus C57BL/6J and Mus musculus molossinus JF1. The Dnmt3L knockout line was maintained on a mixed 129/terSv x C57BL/6 background. Embryos at 9.5 days post coitum (dpc) were obtained from the mating of a Dnmt3L+/+ or Dnmt3L-/- female and a JF1 male. Tail DNA was used for genotyping by PCR as previously described.

Wild animals

Not appliacble

Reporting on sex

Sex was not considered as a biological variable in this study. Brain organoids were generated from male hybrid ESC line, precluding sex comparison for this part of the study. For newborn brain tissue and Dnmt3l embryos, the study focuses on autosomal, parent-of-origin epigenetic regulation of the imprinted Mest/Copg2 locus, a mechanism independent of sex chromosomes and not known to be sexually dimorphic at these developmental stages.

Field-collected samples

Not applicable

Ethics oversight

Animal experiments related to Dnmt3L conceptuses were performed according to a protocol approved by the Institutional Animal Care and Use Committee of the National Center for Child Health and Development (NCCHD) (approval number: 2010–002).
For brain collection from newborn pups, Mus musculus C57BL/6J and Mus musculus molossinus JF1 mouse colonies were maintained and crossed in a certified animal facility (Université Clermont Auvergne, approval number D 63 113 16) in accordance with the European directive 2010/63/EU. Ethical approval was not required under applicable legislation, as the experiments involved the use of post-mortem samples

Note that full information on the approval of the study protocol must also be provided in the manuscript.

Plants

Seed stocks

Report on the source of all seed stocks or other plant material used. If applicable, state the seed stock centre and catalogue number. If plant specimens were collected from the field, describe the collection location, date and sampling procedures.

Novel plant genotypes

Describe the methods by which all novel plant genotypes were produced. This includes those generated by transgenic approaches, gene editing, chemical/radiation-based mutagenesis and hybridization. For transgenic lines, describe the transformation method, the number of independent lines analyzed and the generation upon which experiments were performed. For gene-edited lines, describe the editor used, the endogenous sequence targeted for editing, the targeting guide RNA sequence (if applicable) and how the editor was applied.

Authentication

Describe any authentication procedures for each seed stock used or novel genotype generated. Describe any experiments used to assess the effect of a mutation and, where applicable, how potential secondary effects (e.g. second site T-DNA insertions, mosiacism, off-target gene editing) were examined.

ChIP-seq

Data deposition

- [x] Confirm that both raw and final processed data have been deposited in a public database such as GEO.
- [ ] Confirm that you have deposited or provided access to graph files (e.g. BED files) for the called peaks.

Data access links<br>*May remain private before publication.*	The GEO accession number for Cut & RUN is GSE325583
Files in database submission	GSM9608954 ES BJ H3K4me3 batch1<br>GSM9608955 ES BJ H3K4me3 batch2<br>GSM9608956 NBB BJ H3K4me3 batch1<br>GSM9608957 NBB BJ H3K4me3 batch2<br>GSM9608958 NBB JB H3K4me3 batch1<br>GSM9608959 NBB JB H3K4me3 batch2<br>GSM9608960 ES BJ H3K27ac batch1<br>GSM9608961 ES BJ H3K27ac batch4<br>GSM9608962 NBB BJ H3K27ac batch1<br>GSM9608963 NBB BJ H3K27ac batch2<br>GSM9608964 NBB JB H3K27ac batch1<br>GSM9608965 NBB JB H3K27ac batch2<br>GSM9608966 NBB BJ H3K36me3 batch1<br>GSM9608967 NBB BJ H3K36me3 batch2<br>GSM9608968 NBB JB H3K36me3 batch1<br>GSM9608969 NBB JB H3K36me3 batch2<br>GSM9608970 ES BJ H3K27me3 batch1<br>GSM9608971 ES BJ H3K27me3 batch3<br>GSM9608972 NBB BJ H3K27me3 batch1<br>GSM9608973 NBB BJ H3K27me3 batch2<br>GSM9608974 NBB JB H3K27me3 batch1<br>GSM9608975 NBB JB H3K27me3 batch2<br>GSM9608976 NBB BJ CTCF batch1<br>GSM9608977 NBB BJ CTCF batch2<br>GSM9608978 NBB JB CTCF batch1<br>GSM9608979 NBB JB CTCF batch2<br>GSM9608980 ES BJ IgG batch1<br>GSM9608981 ES BJ IgG batch2<br>GSM9608982 ES BJ IgG batch3<br>GSM9608983 NBB BJ IgG batch1<br>GSM9608984 NBB BJ IgG batch2<br>GSM9608985 NBB JB IgG batch1<br>GSM9608986 NBB JB IgG batch2
Genome browser session<br>(e.g. UCSC)	Bigwig provided in GEO

Methodology

Replicates	Cut&Run experiments were performed in two biological replicates.
Sequencing depth	GSM9608954 ES BJ H3K4me3 batch1 44 100 300 pairs of reads, of which 23 501 222 are aligned concordantly exactly 1 time<br>GSM9608955 ES BJ H3K4me3 batch2 28 491 111 pairs of reads, of which 14 890 895 are aligned concordantly exactly 1 time<br>GSM9608956 NBB BJ H3K4me3 batch1 48 140 419 pairs of reads, of which 31 690 245 are aligned concordantly exactly 1 time<br>GSM9608957 NBB BJ H3K4me3 batch2 29 734 574 pairs of reads, of which 12 359 421 are aligned concordantly exactly 1 time<br>GSM9608958 NBB JB H3K4me3 batch1 26 877 468 pairs of reads, of which 17 813 135 are aligned concordantly exactly 1 time<br>GSM9608959 NBB JB H3K4me3 batch2 18 035 224 pairs of reads, of which 7 358 946 are aligned concordantly exactly 1 time<br>GSM9608960 ES BJ H3K27ac batch1 24 396 058 pairs of reads, of which 17 002 496 are aligned concordantly exactly 1 time<br>GSM9608961 ES BJ H3K27ac batch4 27 935 344 pairs of reads, of which 21 474 583 are aligned concordantly exactly 1 time<br>GSM9608962 NBB BJ H3K27ac batch1 25 950 578 pairs of reads, of which 20 304 037 are aligned concordantly exactly 1 time<br>GSM9608963 NBB BJ H3K27ac batch2 18 998 587 pairs of reads, of which 14 077 575 are aligned concordantly exactly 1 time<br>GSM9608964 NBB JB H3K27ac batch1 33 863 359 pairs of reads, of which 28 219 164 are aligned concordantly exactly 1 time<br>GSM9608965 NBB JB H3K27ac batch2 18 475 258 pairs of reads, of which 13 151 296 are aligned concordantly exactly 1 time<br>GSM9608966 NBB BJ H3K36me3 batch1 24 865 502 pairs of reads, of which 16 347 022 are aligned concordantly exactly 1 time<br>GSM9608967 NBB BJ H3K36me3 batch2 26 023 606 pairs of reads, of which 15 626 391 are aligned concordantly exactly 1 time<br>GSM9608968 NBB JB H3K36me3 batch1 29 783 461 pairs of reads, of which 19 091 907 are aligned concordantly exactly 1 time<br>GSM9608969 NBB JB H3K36me3 batch2 25 474 724 pairs of reads, of which 13 830 638 are aligned concordantly exactly 1 time<br>GSM9608970 ES BJ H3K27me3 batch1 26 372 978 pairs of reads, of which 17 499 490 are aligned concordantly exactly 1 time<br>GSM9608971 ES BJ H3K27me3 batch3 21 616 692 pairs of reads, of which 14 735 286 are aligned concordantly exactly 1 time

GSM9608972 NBB BJ H3K27me3 batch1 34 866 371 pairs of reads, of which 21 359 025 are aligned concordantly exactly 1 time
GSM9608973 NBB BJ H3K27me3 batch2 17 317 118 pairs of reads, of which 12 282 183 are aligned concordantly exactly 1 time
GSM9608974 NBB JB H3K27me3 batch1 23 957 381 pairs of reads, of which 14 332 110 are aligned concordantly exactly 1 time
GSM9608975 NBB JB H3K27me3 batch2 17 541 990 pairs of reads, of which 12 384 860 are aligned concordantly exactly 1 time
GSM9608976 NBB BJ CTCF batch1 60 209 711 pairs of reads, of which 29 503 367 are aligned concordantly exactly 1 time
GSM9608977 NBB BJ CTCF batch2 26 388 776 pairs of reads, of which 13 559 539 are aligned concordantly exactly 1 time
GSM9608978 NBB JB CTCF batch1 29 909 004 pairs of reads, of which 18 862 531 are aligned concordantly exactly 1 time
GSM9608979 NBB JB CTCF batch2 17 737 321 pairs of reads, of which 8 531 465 are aligned concordantly exactly 1 time
GSM9608980 ES BJ IgG batch1 37 172 462 pairs of reads, of which 15 258 695 are aligned concordantly exactly 1 time
GSM9608981 ES BJ IgG batch2 21 645 331 pairs of reads, of which 8 197 175 are aligned concordantly exactly 1 time
GSM9608982 ES BJ IgG batch3 24 110 727 pairs of reads, of which 14 018 805 are aligned concordantly exactly 1 time
GSM9608983 NBB BJ IgG batch1 27 989 315 pairs of reads, of which 10 189 615 are aligned concordantly exactly 1 time
GSM9608984 NBB BJ IgG batch2 29 833 488 pairs of reads, of which 12 752 468 are aligned concordantly exactly 1 time
GSM9608985 NBB JB IgG batch1 23 153 231 pairs of reads, of which 11 241 068 are aligned concordantly exactly 1 time
GSM9608986 NBB JB IgG batch2 27 693 267 pairs of reads, of which 3 851 705 are aligned concordantly exactly 1 time

Antibodies

anti-H3K4me3 Epicypher Mixed monoclonal 13-0041 24213007-97
anti-H3K27ac Abcam Polyclonal Ab4729 1101610-4
anti-CTCF Diagenode Monoclonal C15410210 A2354-00234P
anti-H3K27me3 Diagenode Monoclonal C15410195 A824D
Anti-Rabbit IgY (IgG) Epicypher Polyclonal 13-0042 24213007-87

Peak calling parameters

N/A

Data quality

N/A

Software

paired-end reads were mapped to the mm39 masked genome and hybrid genome using Bowtie2. Alignment filtering was done with samtools (view -f 2 -q 20), and the coverage was obtained with bamCoverage (global coverage --normalizeUsing RPKM --binSize 25; allelic coverage: --binSize 25). The UCSC track collection builder tool was used to overlay allelic coverages for genome exploration.
