# Supplementary material for: The elongation of Mest transcript into MestXL sustains, but does not initiate, the maternal allele bias of its convergent gene Copg2 during neurogenesis
Source: Nat Commun. 2026 Sep 23;17:11045. doi: 10.1038/s41467-026-77877-3 (PMC13691114; doi:10.1038/s41467-026-77877-3)
Supplement: Supplementary file 4 [file 41467_2026_77877_MOESM7_ESM.pdf]

nature portfolio

Peer Review File

The elongation of *Mest* transcript into *MestXL* sustains, but does not initiate, the maternal allele bias of its convergent gene *Copg2* during neurogenesis

Corresponding Author: Dr philippe arnaud

This file contains all reviewer reports in order by version, followed by all author rebuttals in order by version.

Version 0:

Reviewer comments:

Reviewer #1

(Remarks to the Author)
Perillous et al. report an in-depth characterization of the reciprocal expression between the Mest and Copg2 imprinted loci using a new model for brain organoid formation in F1-hybrid mouse ESCs. The study applies a complete series of genomic techniques to monitor the timing of induction of allelic biases starting from ESCs, which exhibit minimal bias of Copg2, to Day21 organoids, which exhibit 75% maternal bias. From these analyses, they conclude that convergent transcription from the paternally expressed MestXL transcript is unlikely to be responsible for maternal bias of Copg2, because the bias originates prior to the induction of MestXL. In contrast, deletion or CRISPRi-induced DNA methylation of the Mest promoter abrogates maternal bias. Capture HiC demonstrates the existence of an insulated domain anchored by a CTCF site specifically on the paternal allele, at the differentiation timepoint when maternal bias in Copg2 expression emerges.

The model that is proposed is that at early stages of differentiation, enhancer blocking via paternal-specific CTCF binding reduces upregulation of Copg2 on the paternal allele, followed by, at later stages, patneral-specific repression of Copg2 via convergent transcription/transcriptional collision mediated by MestXL.

The model is in general well justified based on the data presented, with some requests for clarification and potentially additional analyses below. The application of the novel organoid system to study imprinting coupled with the genetic and genomic assays was exciting and powerful.

1) My largest question arises from the stated paternal-specific binding of CTCF to the Mest ICR. This binding is never adequately shown although this may be a situation in which the authors have seen the data themselves but not presented it in a form that was clear to this reviewer.

In Supplemental Figure 4A, I see that the authors performed a DNA methlation analysis to demonstrate paternal-specific unmethylated DNA at sites within the ICR.

Separately, if I squint at the blue CTCF track in Figure 4C, I can detect what appears to be the paternal-specific CTCF peak that sits near the boundary of the insulated domain. However, that peak appears to be outside of the ICR?

Given the intriguing model, the outstanding allelic data that the authors have amassed, and its importance to model, I request that the authors move Supplemental Figure 4A as a main figure and show a much zoomed in version of the allele-specific CTCF data that is currently in Figure 4C as part of a new figure panel that enables me to evaluate as a reviewer whether the location of the parent of origin specific CTCF binding coincides with the DMR/ICR.

Minor comments:

2) The fonts in all figures were very small and made it difficult to interpret the article on a printed page, to the detriment of my understanding.

3) I appreciated seeing the data in supplemental Figures 2A and 2B and felt that it would warrant inclusion in the main figure

if the authors agreed.

4) In line 255 -- as a reader, I need more help understanding how the results provide compelling evidence of imprinting. Wouldn't that require a reciprocal cross? If this interpretation is correct, softening the statement might be warranted. If I misunderstood, then additional explanation would be helpful.

5) as a thought for consideration, I noticed that Mest upregulation at D7 coincides with maternal bias of Copg2 -- is it possible that transcriptional induction of Mest acts as an enhancer blocking mechanism that potentially loads CTCF and prevents maternal up regulation? or if CTCF is not bound to the actual ICR (major request #1), then perhaps the promoter activation model could be invoked as a mechanism of enhancer blocking.

Reviewer #2

(Remarks to the Author)
The elongation of Mest transcript into MestXL sustains, but does not initiate, the maternal allele bias of its convergent gene Copg2 during neurogenesis

The authors have used a number of highly sophisticated manipulations of mouse embryonic stem cells differentiated in vitro to brain organoids to tease apart the mechanism resulting in constitutive paternal expression of Mest (Peg1) and the transition from biallelic to maternal bias of Copg2 as the central nervous system develops. Studies on a number of imprinted domains such as Igf2r and KvDMR implicate long non-coding RNAs as mediators of long range imprinting signals either through a process of direct transcriptional interference or potentially through recruitment of epigenetic modifiers. The authors demonstrate that the long non-coding RNA MestXL initiates at the same promoter as Mest, and further demonstrate that premature termination of MestXL leads to loss of maternal biased Copg2. Through very precise multi ‘omic and temporal analysis, the maternal allele bias of Copg2 is detectable in advance of upregulation of MestXL expression through the domain. This suggests that the maternal biased expression is established by a different mechanism and only maintained by MestXL which is an exciting discovery that may be replicated at other loci when these are similarly investigated at this detailed level. Overall, the experimental approaches are well designed and clearly described, significant work has been undertaken and the findings are robust. The work answers some important questions and also raises new questions that are not all answered. MestXL is required from the maintenance of maternal-bias but not the initiation. The initiating mechanism has not been identified despite extensive investigation.

One key question is whether the dosage of Copg2 is changing over development due to the allelic changes or simply that there are different levels of expression of Copg2 as cells differentiate. The data is in the figures but hard to visualise. For example, although total expression is presented relative to a fixed value across the three transcripts in Figure 1, a separate column is presented for Copg2 showing how the % allele changes as expression changes through differentiation. It appears that the expression level of Copg2 is not changing simply due to increased maternal allele expression or decreased paternal allele expression. The combined data ie expression/allele is in the genome browser view but hard to visualise. Findings presented in figure 3 suggest persistence of paternal Copg2 is associated with overall higher Copg2 expression. Is this correct? I feel that these data should be illustrated more explicitly throughout as I would like to see how the expression of each allele changes over time since this supports the interpretation of the findings.

It will also be important to strengthen the argument that these studies are functionally important. Bcl2l1 is mentioned as an example where parental bias is important and it would be helpful to have ratio here to provide more context to the reader. The authors mention that human MEST has been linked to cognitive ability, autism susceptibility and neurodevelopmental disorders and give the example of a microdeletion in SRS but what are the other mutations reported? Similarly, can the authors add a little more detail on genetic alterations affecting COPG2 associated with neurodevelopmental disorders. Are these inactivating mutations? heterozygous? On maternal allele exclusively? This is critically important since the switch from biallelic to maternal bias is subtle ie from 50:50 to 70:30. To really elevate the findings, the functional relevance is critical.

Did the authors undertake analyse the cellular composition of their brain organoids in the different models? The assumption is that changes in expression reflect increases/decreases in expression in the manipulated models with transcripts co-expressed in the same cells but could there be differences in cell types expressing the various transcripts?

Figure 1B Looks like the peaks are cut off for Day 7 and 14. Quite hard to see what is going on.

Figure 1C. To clarify does “percentage of expression relative to the geometric mean of three housekeeping genes” means that – for example – Day 7 MEST is approx. 1.3%, Day 7 Copg2 is approx. 0.17%. Presume this intended to give an idea of relative levels between the different transcripts?

Reviewer #3

(Remarks to the Author)
Genomic imprinting continues to provide interesting paradigms on the epigenetic regulation of gene expression and allele-specific gene expression seems to be more complex to untangle than previously appreciated, since imprinted genes do not all neatly fit into either the ICR insulator or the reciprocal sense antisense inhibition paradigms.

The current study investigates how the mouse Copg2 gene switches from biallelic expression to allele-specific expression during neural lineage specification. The imprinted Mest/Copg2 locus is clinically important and has been linked to Silver Russel syndrome, cognitive ability, autism and neurodevelopment disorders. This locus is also interesting in that it has a tissue specific polyadenylation that results in a long transcript that inhibits the neighbouring Copg2 locus. Coordinate gene expression and transcriptional inhibition is still a relatively poorly understood topic.

The strengths of the current study is that the authors have used hybrid ESC cells derived from reciprocal C57/black6 x JF1 mice to establish a mouse brain organoid system. The authors were able to do differential stage specific analyses of the Mest/Copg2 domain. This is a powerful experimental system, that can be manipulated with CRISPRi and CRIPR-mediated KO genome editing. Insertion of poly adenylation signals is particularly elegant :) Further strengths include that the expression patterns of Mest/Copg2 in the differentiated organoids mirror that of newborn mice.

Specific suggestions regarding background and concepts presented in the introduction
1. It has been suggested by several authors over the years that imprinting is a form of gene dosage, yet in the same papers, they present mono allelic expression data that show higher levels of expression than biallelic expression. If you going to say imprinting is important dosage compensation mechanism, then qualify how for the Mest/Copg2 biallic expression leads to pathology.
2, Take into consideration the wider audience who are not familiar with the locus and explain with a figure what the locus looks like in mice provide a simplified initial figure detailing the direction of allele-specific and biallelic transcription, location of promoters, alternative transcripts etc the ICR, other potential sites that have potential regulatory functions (eg CTCF sites). Although this information can be gleaned from the browser screenshots provided in the figures, it is still helpful to orientate the reader from the beginning.
Specific comments/suggestions regarding the data and presentation of results
1. Can the Seq-smiFISH data be quantified? This also seems to be a powerful tool and a pity that it wasnt used more extensively throughout the study to show mono and biallelic signals of Copg2 in addition to Mest isoforms.
2. I find it both curious and interesting that the CRISPRi-ICR simultaneously blocked transcription and methylated the ICR. Can the authors provide a reason why this happens? (Blocking Zgp57 binding site? Other reasons?)
3. It is obviously good to have a data for E9.5 mouse brains, but for better comparison to the "maternalised" ICR, it would have been nice to see organoids derived from Dmnt3l KO ESCs. Perhaps if the description of the results started with the description of the "paternalised" ICR in the Dnmt3l mice, and then introduced the strategy to test the "maternalised"mice, this section could flow better.
4. Given the relationship between MestXL, Mest and Copg2, it would be good to show the effect of the fully unmethylated ICR on overall expression of Mest and MestXL in Figure 3E. Although the suppl figure shows a heatmap, I would have preferred to see quantitative RT-PCR expression levels of Mest and MestXL together with the Copg2 data in this figure to compare between WT and Dmnt3L-/+ embryos. (The claim made in this fig is that the ICR is important for the allelic switch, thus its effects on the Mest and MestXL should be included here, not in Suppl data).
5. The resolution of the CTCF profiles produced in Fig 4C isn't good enough to see the exact position of the peaks. Consider showing this separately or part of Fig 4B. Fig 4B could benefit from including the Mest promoter and also highlighting the ICR region so that the reader can orientate where the various histone modifications are relative to CTCF binding sites and the allele specific CTCF at the ICR.
6. The HIC contact matrices could have been explained better in the text, especially the very striking allele specific stripes should be pointed out and where they are relative to the Mest and Copg2 promoters. These stripes may provide clues to the locations of potential enhancer elements that the Copg2 promoter could access.
7. Interesting result showing that MestXL doesn't mediate allele-specific expression. It's difficult to call a an allele-specific bias based on allele-specific ratios. Depending on the assay, biallelic expression without allele bias can give read outs between 50-65, which suggests that 70% is the threshold for allele bias. Also, the allelic ratios do look a bit different between WT and pAS at days 14 and 21. At day 21 it also seems as if the levels of Copg2 is increased in the pAS compared to WT. Why not present the data as WT compared to pAS on the same graph for more direct comparison. This is also another instance where the FISH analysis would have been good to determine whether the proportion of cells with monoallelic, or biallelic expression.

Version 1:

Reviewer comments:

Reviewer #1

(Remarks to the Author)
I would like to thank the authors for their thoughtful responses to my questions, and congratulate them on what I feel is a rigorous and intriguing study.

Reviewer #2

(Remarks to the Author)
Thank you for the clear responses and amendments.

Reviewer #3

(Remarks to the Author)
I am satisfied that the authors have addressed all of my comments. I like the new Figure 1 explaining the locus and the improved figure legends. It is a pity that smiFISH could not be applied to Copg2 but I appreciate the technical difficulties in optimising this.

Open Access This Peer Review File is licensed under a Creative Commons Attribution 4.0 International License, which permits use, sharing, adaptation, distribution and reproduction in any medium or format, as long as you give appropriate credit to the original author(s) and the source, provide a link to the Creative Commons license, and indicate if changes were made.
In cases where reviewers are anonymous, credit should be given to 'Anonymous Referee' and the source.
The images or other third party material in this Peer Review File are included in the article's Creative Commons license, unless indicated otherwise in a credit line to the material. If material is not included in the article's Creative Commons license and your intended use is not permitted by statutory regulation or exceeds the permitted use, you will need to obtain permission directly from the copyright holder.
To view a copy of this license, visit https://creativecommons.org/licenses/by/4.0/

We are grateful to the three reviewers for their thorough and constructive evaluation of our manuscript. Their comments have led us to clarify key conceptual points, strengthen the mechanistic interpretation, and improve the presentation of several figures. We have carefully addressed all requests and revised the manuscript accordingly. Each point is addressed in detail below, with reviewer comments reproduced in italics, followed by our response and a description of the corresponding changes to the manuscript.

To facilitate the review process, we have provided, alongside the revised manuscript, a supplemental file entitled "highlighted revised ms" in which all modifications relative to the initial submission are indicated in red.

Reviewer 1

We thank Reviewer 1 for recognising the power of our organoid model and the quality of our allele-specific genomic data, and for highlighting points that needed clarification regarding the allelic CTCF binding at the *Mest* ICR, which is central to the proposed model.

Major comment – Point 1: Allele-specific CTCF binding at the Mest ICR and its relationship to the DMR

> *My largest question arises from the stated paternal-specific binding of CTCF to the Mest ICR. This binding is never adequately shown. In Supplemental Figure 4A, I see that the authors performed a DNA methylation analysis to demonstrate paternal-specific unmethylated DNA at sites within the ICR. Separately, if I squint at the blue CTCF track in Figure 4C, I can detect what appears to be the paternal-specific CTCF peak that sits near the boundary of the insulated domain. However, that peak appears to be outside of the ICR? I request that the authors move Supplemental Figure 4A as a main figure and show a much zoomed in version of the allele-specific CTCF data currently in Figure 4C as part of a new figure panel.*

Response:

We thank the reviewer for raising this important point. We agree that the allele-specific CTCF binding and its spatial relationship to the DMR/ICR were not presented with sufficient clarity. We have therefore introduced a new panel in the revised Figure 6 and made the following changes in the revised manuscript:

- We have added a new, high-resolution zoomed-in genome browser panel centred specifically on the *Mest* promoter/ICR region, displaying allele-specific CTCF and histone mark CUT&RUN signals from newborn brain tissue alongside the positions of the CpG island and ICR, as well as publicly available WGBS tracks from ESCs and NPCs (revised Fig.6a).

- In addition to the WGBS data presented in new Fig. 6a, we now provide in new Fig. 6b bisulfite sequencing data directly demonstrating the allelic methylation pattern at the *Mest* ICR in neonatal brain tissue.

These panels unambiguously demonstrate that the paternal-specific CTCF peak is located precisely within the boundaries of the *Mest* ICR, overlapping the unmethylated paternal CpG island/promoter. This is consistent with the well-established methylation-sensitive binding of CTCF at imprinting control regions (Prickett et al., 2013, ref 11). We note that in the previous version, the CTCF peak could appear to fall slightly outside the ICR owing to the scale of the genome browser view; the zoomed panel now resolves this ambiguity.

Minor comment – Point 2: Font size in figures

> *The fonts in all figures were very small and made it difficult to interpret the article on a printed page.*

Response:

We apologise for this issue. All figures have been revised with larger font sizes for axis labels, tick marks, gene annotations, and legends to improve readability on a printed page.

Minor comment – Point 3: Supplemental Figures 2A and 2B to be included in the main figures

> *I appreciated seeing the data in supplemental Figures 2A and 2B and felt that it would warrant inclusion in the main figure if the authors agreed.*

Response:

We agree with the reviewer that these data are important for the narrative. However, we are also constrained by the number of panels that can be incorporated into a single figure while maintaining sufficient readability for the reader. To best address this point, the long-read RNA-seq data (formerly Supplemental Figure 2a, supporting that *MestXL* initiates from the *Mest* promoter) have been integrated into Figure 3 (Fig.3a) of the revised manuscript (formerly figure 2). Meanwhile, the RT-qPCR analyses in CRISPRi lines, which are complementary to the RNA-seq data presented in Figure 3d, have been retained in Supplemental Figure 3a.

Minor comment – Point 4: Strength of the imprinting evidence (line 255)

> *As a reader, I need more help understanding how the results provide compelling evidence of imprinting. Wouldn't that require a reciprocal cross? If this interpretation is correct, softening the statement might be warranted.*

Response:

The reviewer raises an important point that was not presented with sufficient clarity in the original manuscript. To confirm that an observed event is genuinely linked to imprinting, it is indeed necessary to track it across reciprocal crosses, which is precisely what we do in this section. The data presented in Figure 4 d, g of the revised manuscript were obtained from the B/J line, while those in supplemental Figure 4 e were derived from the reciprocal J/B line.
The use of reciprocal F1 hybrid backgrounds (B/J and J/B) provides critical evidence for the parent-of-origin nature of this regulation: the maternal allele bias observed in controls is consistently lost under CRISPR-ICR conditions, regardless of which parental strain contributes the maternal allele.
To ensure this is immediately apparent to the reader, we have now explicitly stated the genotype of the cells used in Figure 4 directly in the main text, previously this information appeared only in the figure legend, and have revised the final sentence of this results section accordingly.

Minor comment – Point 5: Transcriptional induction of Mest at D7 as a potential CTCF-loading / enhancer-blocking mechanism

> *I noticed that Mest upregulation at D7 coincides with maternal bias of Copg2 – is it possible that transcriptional induction of Mest acts as an enhancer blocking mechanism that potentially loads CTCF and prevents maternal upregulation? or if CTCF is not bound to the*

> *actual ICR (major request 1), then perhaps the promoter activation model could be invoked as a mechanism of enhancer blocking.*

Response:

As now clarified (Point 1 above), paternal CTCF binding coincides with the ICR/*Mest* promoter, supporting the conclusion that promoter activation alone cannot account for enhancer blocking in this context. That said, the reviewer's suggestion that activation of the canonical *Mest* promoter at D7, evidenced by the progressive gain of H3K9ac and H3K27ac marks, (Fig.3 c) may stabilise or reinforce CTCF occupancy on the paternal allele is an excellent one, consistent with the known interplay between active transcription and CTCF binding dynamics. Under this model, enhanced paternal promoter activity would consolidate the insulating chromatin architecture at the ICR, directing upstream enhancer activity preferentially toward the maternal *Copg2* allele. This is now discussed in a new dedicated paragraph in the revised Discussion, alongside recent independent support from Bae et al. (ref. 41), who identified a ~150 kb upstream enhancer with differential allelic activity at this locus.

Reviewer 2

We thank Reviewer 2 for the positive assessment of our experimental approaches and for suggestions that help to clarify the biological significance of our findings and improve the presentation of the allelic expression data.

Point 1: Explicit visualisation of allele-specific expression levels over time

> *One key question is whether the dosage of Copg2 is changing over development due to the allelic changes or simply that there are different levels of expression of Copg2 as cells differentiate. I feel that these data should be illustrated more explicitly throughout as I would like to see how the expression of each allele changes over time since this supports the interpretation of the findings.*

Response:

We agree that the separate allelic trajectories of *Copg2* expression are central to our interpretation and were insufficiently highlighted in the original manuscript. We have therefore added new panels to Figures 2, 4 and 7 showing the estimated expression levels of the maternal and paternal *Copg2* alleles separately at each differentiation stage, derived by applying the RNA-seq allelic ratios to total RPKM values. This revised representation strengthens our conclusions by making explicit that:

- At Day 7, the maternal allele bias results primarily from an upregulation of the maternal allele (consistent with enhancer-driven activation);
- At Days 14 and 21 in WT cells, overall *Copg2* levels decline, but the maternal bias is maintained and reinforced by relative repression of the paternal allele (consistent with MestXL-mediated transcriptional interference);
- In the pAS line, the absence of *MestXL* prevents this paternal repression, resulting in an increased paternal allele transcript level at later stages. This explains the elevated total *Copg2* levels observed at Day 21.

Point 2: Functional relevance – details on MEST and COPG2 mutations in human neurodevelopmental disorders

> *It will also be important to strengthen the argument that these studies are functionally important. The authors mention that human MEST has been linked to cognitive ability, autism susceptibility and neurodevelopmental disorders and give the example of a microdeletion in SRS but what are the other mutations reported? Similarly, can the authors add a little more detail on genetic alterations affecting COPG2 associated with neurodevelopmental disorders. Are these inactivating mutations? heterozygous? On maternal allele exclusively? This is critically important since the switch from biallelic to maternal bias is subtle ie from 50:50 to 70:30.*

Response:

This is an important point, and we have substantially expanded this section of the Introduction.

Regarding *MEST*, we now detail that the 7q32.2 microdeletions associated with Silver-Russell syndrome-like features are paternally inherited heterozygous deletions (22, 23), directly confirming the dosage-sensitivity of the paternal *MEST* allele. Beyond structural variants, *MEST* promoter methylation in adult blood has been associated with childhood cognitive ability in two independent longitudinal cohorts (n = 485 and n = 223), implicating *MEST* imprinting as a mechanistic contributor to the heritable component of cognitive function (20). Paternally inherited cis-regulatory structural variants encompassing *MEST* are also preferentially transmitted to children with autism spectrum disorder (new added reference :Brandler et al., 2018), consistent with the parent-of-origin specificity of *MEST* expression.

Regarding *COPG2*, we now note that the variants identified by Sanders et al. (25) in ASD include de novo predicted loss-of-function variants. However, as explicitly stated in the revised text, the parental origin of these variants has not been systematically reported — a critical gap given that *COPG2* is preferentially expressed from the maternal allele in the developing brain.

Regarding the functional significance of the 50:50 to 70:30 allelic shift, we draw on the well-characterized example of *Bcl2l1*, where a modest paternal bias (60:40) in brain carries clear functional consequences: paternal-specific deletion, but not maternal, leads to loss of specific neuronal subtypes (5), demonstrating that even moderate allelic imbalances can drive discrete phenotypic outcomes. This directly supports the functional relevance of the maternal *COPG2* bias documented here, a point we now explicitly stress in the revised Discussion.

Point 3: Cellular composition of brain organoids across models

> *Did the authors undertake analyses of the cellular composition of their brain organoids in the different models? The assumption is that changes in expression reflect increases/decreases in expression in the manipulated models with transcripts co-expressed in the same cells but could there be differences in cell types expressing the various transcripts?*

Response:

We thank the reviewer for raising this important point, which we now address explicitly. RT-qPCR profiling of lineage markers had previously demonstrated comparable differentiation trajectories between WT and CRISPRi-ICR lines (Supplemental Figure 4c). To consolidate these data and extend the analysis to the pAS model, we compared the expression of neural lineage markers across all three models, WT, CRISPRi-ICR, and pAS, at all differentiation stages, using RNA-seq data. Markers assessed encompassed pluripotency (*Nanog* and *Pou5f1*), proliferation (*Mki67*), neural/cortical progenitor (*Nestin, Fabp7, Sox1, Pax6, Emx1, and Foxg1*), neuronal/cortical neurons (*Elavl4, Dcx, Tbr1, Tubb3, Tbr1, Bcl11b, Sox5, Pou3f2, and Tox)*, astrocyte (*Gfap*) and oligodendrocyte (*Olig1* and *Olig2*). The overall differentiation trajectories are comparable across all three models, with no major shifts in markers of cell identities at any stage. These new data are presented in Supplemental Figures 4d and 6c, respectively, and are described in the corresponding Results sections.

Points 4 & 5: Figure 1B peak truncation

> *Figure 1B: Looks like the peaks are cut off for Day 7 and 14. Quite hard to see what is going on.*

Response:

We thank the reviewer for this observation. The peaks in Figure 2b (formerly Figure 1) are indeed truncated at Day 7, a deliberate choice driven by the large difference in expression levels between Day 7 and the other stages. The purpose of this panel is to convey the allelic dynamics and overall expression trends of *Mest*, *MestXL*, and *Copg2* across the differentiation time course, rather than absolute signal amplitude at each stage. An alternative version scaled to the full Day 7 signal, without peak truncation, is shown below for reference.

While this untruncated version better illustrates the magnitude of the *Mest* expression peak at Day 7, it comes at the cost of resolution at the other stages, making it difficult to appreciate the emergence of the *Copg2* allelic bias and the onset of *MestXL* induction. We therefore favoured the truncated representation, which preserves visibility of these allelic dynamics at every stage. The quantitative differences in expression levels between the three genes are in any case fully captured in panels c–f of the same figure, which should be considered together with panel b for a complete picture.

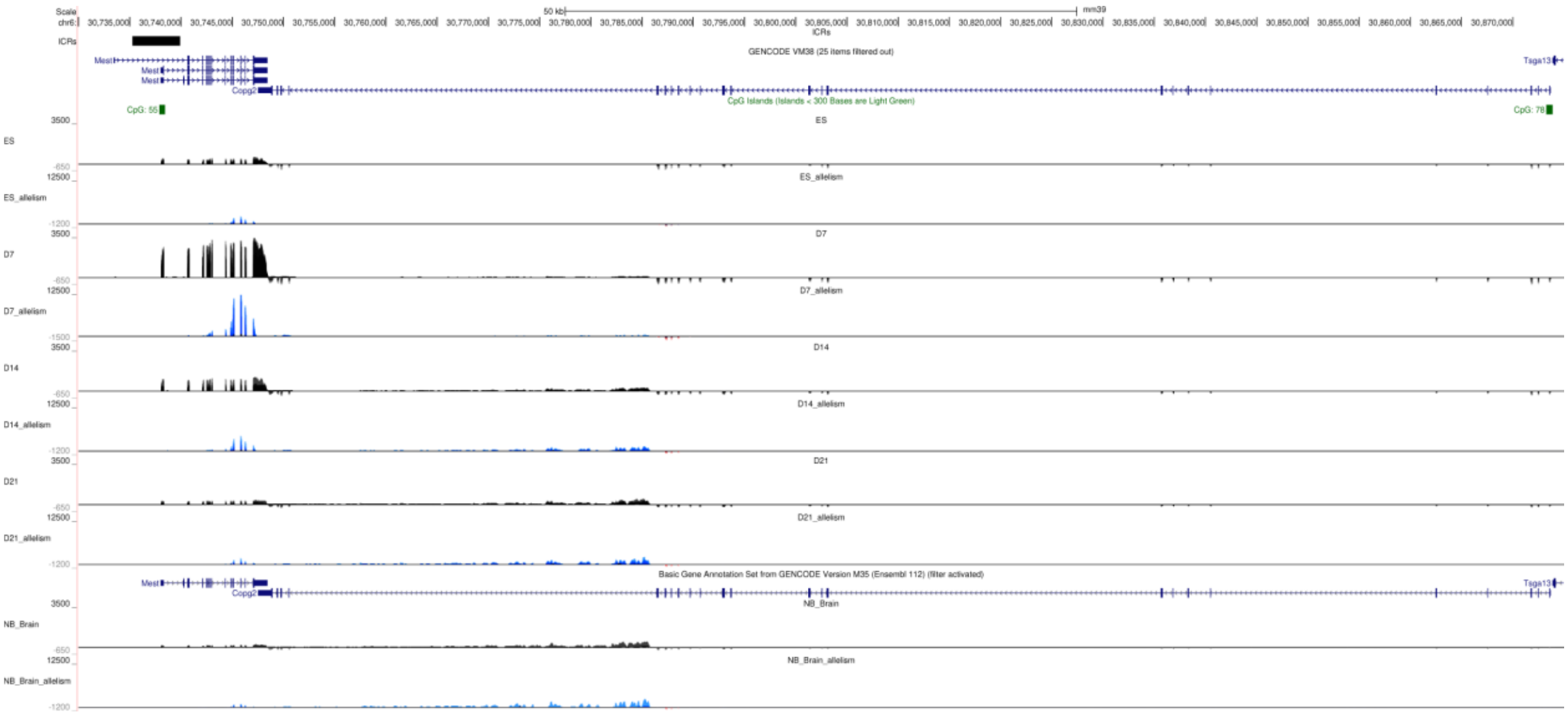

Point 5: direct WT vs. pAS comparison

> *Figure 1C: To clarify does "percentage of expression relative to the geometric mean of three housekeeping genes" mean that – for example – Day 7 MEST is approx. 1.3%? Presume this is intended to give an idea of relative levels between the different transcripts?*

Yes, the values represent the expression level of each transcript as a percentage of the geometric mean of the three housekeeping genes (*Gapdh*, *Gus*, *Tbp*), enabling quantitative comparison of relative transcript levels between *Mest*, *MestXL*, and *Copg2* across stages. This is now stated explicitly in the figure legend.

Reviewer 3

We thank Reviewer 3 for the positive and detailed assessment and for constructive suggestions that improve both the accessibility of the manuscript and the depth of the mechanistic interpretation.

Background/Introduction – Point 1: Qualification of imprinting as a dosage mechanism

> *It has been suggested by several authors over the years that imprinting is a form of gene dosage, yet in the same papers, they present monoallelic expression data that show higher levels of expression than biallelic expression. If you are going to say imprinting is important as a dosage compensation mechanism, then qualify how biallelic Copg2 expression leads to pathology.*

Response:

The reviewer raises an important conceptual point. We have clarified in the Introduction (second paragraph) that genomic imprinting is not a dosage compensation mechanism in the classical sense, but rather a system of precise dosage control in which the quantitative output from a single parental allele is tightly calibrated. Framing genomic imprinting as a simple "halving" of gene dosage is indeed an oversimplification. The output from the active parental allele is not a fixed quantity but is subject to tissue-specific and developmental-stage-specific tuning, a dynamic calibration that is critical for establishing cellular identity.

It is also important to clarify that biallelic *Copg2* expression is not in itself pathological: *Copg2* is biallelically expressed throughout most of embryonic development, and it is only in neural tissues, at a precise developmental window, that allelic usage shifts toward preferential maternal expression. The pathological risk therefore does not arise from biallelic expression per se, but from the failure of this expected allelic switch, through loss of imprinting, and the consequent breakdown of precise dosage control in a specific tissue and temporal context.

Our study illustrates this principle directly: we show that *Copg2* levels are tightly regulated during neurogenesis through imprinting mechanisms, first increasing in neural progenitors and subsequently declining at later neuron-enriched stages. This two-step regulation, which we uncover here, exemplifies how imprinting does not simply restrict expression to one allele, but orchestrates a precise quantitative output whose disruption at the wrong moment, in the wrong tissue, may carry functional consequences.

Background/Introduction – Point 2: Schematic figure of the locus

> *Take into consideration the wider audience who are not familiar with the locus and explain with a figure what the locus looks like in mice: a simplified initial figure detailing the direction of allele-specific and biallelic transcription, location of promoters, alternative transcripts, the ICR, other potential regulatory sites (e.g. CTCF sites).*

Response:

To improve the accessibility of our manuscript, we have added a new figure, Figure 1, in the revised manuscript, providing a schematic diagram of the *Mest*/*Copg2* convergent imprinted gene pair. This schematic depicts the positions and orientations of *Mest* and *Copg2*, the location of the maternally methylated ICR, the relevant promoters, and the known allelic expression profiles in neural and non-neural tissues.

Data/Results – Point 1: Quantification of Seq-smiFISH data

> *Can the Seq-smiFISH data be quantified? This also seems to be a powerful tool and a pity that it wasn't used more extensively throughout the study to show mono and biallelic signals of Copg2 in addition to Mest isoforms.*

Response:

As requested by the reviewer, we have now included the quantification of the Seq-smiFISH data. Using a state-of-the-art analysis pipeline (see *Methods*), we have quantified the percentage of nuclei displaying *Mest* transcription sites in CRISPRi-control and CRISPRi-ICR organoids (Revised Supplementary Figure 3b, e), as well as the percentage of nuclei displaying co-localisation of the three probe sets (*Mest* intron 1/ *MestXL* proximal/ *MestXL* distal) (revised Supplemental Figure 3c, f). This analysis confirms that *Mest* is monoallelic (in 11% of cells) and the triple colocalisation of *Mes*t intron1/ with *MestXL* proximal and distal probes (in 12% of cells positive for *Mest* intron 1).

We share the Reviewer's enthusiasm for using Seq-smiFISH to interrogate the allelism of *Copg2*. However, our preliminary data suggest that this approach requires significant technical development before it can be applied meaningfully to *Copg2*. Unlike *Mest*, where a single intronic probe close to the transcription start site is sufficient to detect nascent monoallelic transcription, a comprehensive allelic analysis of *Copg2* is more demanding. One mechanistic possibility is that *MestXL* impacts *Copg2* allelism through collision between RNA Pol II complexes transcribing in antisense directions, which would result in premature termination and shorter *Copg2* transcripts rather than suppression of transcription initiation. Under this model, detection of the *Copg2* transcription site by a single proximal intronic probe would be insufficient to capture the full extent of allelic regulation, and a comprehensive analysis would require designing a series of intronic probes spanning the entire *Copg2* transcript length. We are currently developing such probe sets, but we believe that these ongoing experiments, which are likely to be the foundation of a future dedicated study, fall outside the scope of the present manuscript.

Data/Results – Point 2: CRISPRi-ICR simultaneous transcriptional blocking and methylation of both alleles

> *I find it both curious and interesting that the CRISPRi-ICR simultaneously blocked transcription and methylated the ICR. Can the authors provide a reason why this happens?*

Response:

We thank the reviewer for this question. The gain of DNA methylation at CpG island promoters following dCas9-KRAB-MeCP2 targeting is documented in Ding et al. (2022), who demonstrated by bisulfite sequencing that this construct induces de novo DNA methylation at endogenous promoters in mESCs, the same cellular context as our study. We have now included this reference in the "CRISPR-Cas9 inhibition" Material and Methods section.
Mechanistically, the KRAB domain of the dCas9-KRAB-MeCP2 fusion (Yeo et al., 2018) recruits SETDB1, promoting local heterochromatin formation through H3K9 trimethylation and HP1 deposition. This repressive chromatin environment favours, in addition to MeCP2 domain, the recruitment of de novo DNA methyltransferases DNMT3A and DNMT3B to the targeted locus, resulting in the acquisition of DNA methylation.

Data/Results – Point 3: Presentation order – Dnmt3l before CRISPRi-ICR

> *It is obviously good to have data for E9.5 mouse brains, but for better comparison to the 'maternalised' ICR, it would have been nice to see organoids derived from Dnmt3l KO ESCs.*

> *Perhaps if the description of the results started with the description of the 'paternalised' ICR in the Dnmt3l mice, and then introduced the strategy to test the 'maternalised' mice, this section could flow better.*

Response:

We agree that organoids derived from Dnmt3l KO ESCs would be an elegant complementary system to model the "paternalised" ICR context in vitro. Dnmt3l-deficient mESC lines derived from blastocyst-stage embryos have indeed been described (Ge et al., 2019, PMID: 30321403). However, two limitations preclude their use in our study. First, these lines were not generated in a hybrid genetic background, which is a prerequisite for the allele-specific analyses that underpin our study. Second, and more critically, Ge et al. reported that Dnmt3l mKO ESCs exhibit impaired differentiation capacity, with obvious defects in embryoid body formation under standard differentiation conditions.

We also agree with the reviewer that restructuring the Results section improves the logical flow of the manuscript. In the revised version (results section and new figure 4), we now present first the Dnmt3l$^{-/+}$ embryo data, which represent the "paternalised" ICR context, followed by the CRISPRi-ICR data, which model the complementary "maternalised" ICR context in vitro. This ordering makes the mechanistic interpretation more intuitive for the reader.

Data/Results – Point 4: Include Mest and MestXL expression in Figure 3E (Dnmt3l embryos)

> *Given the relationship between MestXL, Mest and Copg2, it would be good to show the effect of the fully unmethylated ICR on overall expression of Mest and MestXL in Figure 3E. Although the suppl figure shows a heatmap, I would have preferred to see quantitative RT-qPCR expression levels of Mest and MestXL together with the Copg2 data in this figure.*

Response:

We agree. We have added quantitative expression data for *Mest* and *MestXL* (assessed via the *Gm56999* transcript annotation) in WT and *Dnmt3l*−/+ E9.5 embryos to Figure 4 (revised panel 4c), alongside the *Copg2* data. This directly illustrates the consequence of ICR demethylation (paternalisation) on all three transcripts simultaneously: upregulation of *Mest*, modest induction of *MestXL*, and concomitant decrease of *Copg2* with loss of maternal allele bias.

Data/Results – Point 5: Resolution of CTCF profiles in Figure 4C; integration with Figure 4B

> *The resolution of the CTCF profiles produced in Fig 4C isn't good enough to see the exact position of the peaks. Consider showing this separately or part of Fig 4B. Fig 4B could benefit from including the Mest promoter and also highlighting the ICR region so that the reader can orientate where the various histone modifications are relative to CTCF binding sites and the allele-specific CTCF at the ICR.*

Response:

This point is directly addressed by the revisions described in response to Reviewer 1, Point 1. We agree that the allele-specific CTCF binding and its spatial relationship to the DMR/ICR were not presented with sufficient clarity. We have therefore introduced a new panel in the revised Figure 6 and made the following changes in the revised manuscript:

- We have added a new, high-resolution zoomed-in genome browser panel centred specifically on the *Mest* promoter/ICR region, displaying allele-specific CTCF and

histone mark CUT&RUN signals from newborn brain tissue alongside the positions of the CpG island and ICR, as well as publicly available WGBS tracks from ESCs and NPCs (revised Fig.6a).

- In addition to the WGBS data presented in new Fig. 6a, we now provide in new Fig. 6b bisulfite sequencing data directly demonstrating the allelic methylation pattern at the *Mest* ICR in neonatal brain tissue.

These panels unambiguously demonstrate that the paternal-specific CTCF peak is located precisely within the boundaries of the *Mest* ICR/gDMR, overlapping the unmethylated paternal CpG island/promoter.

Data/Results – Point 6: Hi-C contact matrices – better explanation of allele-specific stripes

> *The HIC contact matrices could have been explained better in the text, especially the very striking allele-specific stripes should be pointed out and where they are relative to the Mest and Copg2 promoters. These stripes may provide clues to the locations of potential enhancer elements that the Copg2 promoter could access.*

Response:
We have expanded the description of the Hi-C data in the corresponding Results section paragraph. We clarify that the *Mest* promoter lies at the upstream edge of the paternal subdomain boundary, placing it within the upstream subdomain, while the *Copg2* promoter is confined to the downstream subdomain. We further note that this architecture is compatible with the recently identified upstream enhancer (~150 kb from *Mest*, ref. 41): its position within the upstream paternal subdomain anchored by the CTCF-bound ICR provides a structural basis for its allele-specific activity.

Data/Results – Point 7: Threshold for allelic bias; WT vs. pAS comparison; FISH for Copg2

> *It's difficult to call an allele-specific bias based on allele-specific ratios. Depending on the assay, biallelic expression without allele bias can give read outs between 50–65, which suggests that 70% is the threshold for allele bias. Also, the allelic ratios do look a bit different between WT and pAS at days 14 and 21. At day 21 it also seems as if the levels of Copg2 is increased in the pAS compared to WT. Why not present the data as WT compared to pAS on the same graph for more direct comparison?*

Response:

We thank the reviewer for this methodological point. In our WT ESC controls (biallelic reference), the maternal ratio for *Copg2* ranges from 50–56% across replicates, consistent with the expected baseline range for biallelic expression. The values at Day 7 (66–68% in both WT and pAS) are statistically significantly above this biallelic range (ANOVA with post-hoc Tukey test, $p < 0.05$). The subsequent divergence between WT (reaching 73%) and pAS (declining to 60%) at Day 21 is likewise statistically significant ($p < 0.05$), and the allelic ratio differences between WT and pAS at Days 14 and 21 are consistent with a role for *Mest*XL in establishing allelic bias at these stages. These thresholds are now explicitly stated in the corresponding results section paragraph.
Regarding the elevated *Copg2* levels in pAS at Day 21: as now clarified by the allele-specific level analysis presented in the revised Figure 7 (formerly Figure 5) (see response to Reviewer 2, Point 1), this reflects increased stability of the paternal *Copg2* transcript in the absence of

*Mest*XL. As requested, WT and pAS allelic data are now superimposed on the same graphs in the revised Figure 7 (formerly Figure 5).

We hope that the revised manuscript and these responses address all concerns raised by the reviewers. We remain available to provide any additional clarification or data that the Reviewers may require.
